# Handgrip Strength Test and Bioelectrical Impedance Analysis in SARS-CoV-2 Patients Admitted to Sub-Intensive Unit

**DOI:** 10.3390/nu15081979

**Published:** 2023-04-20

**Authors:** Sonia Zotti, Isabella Luci, Panaiotis Finamore, Francesco Travaglino, Claudio Pedone, Raffaele Antonelli Incalzi

**Affiliations:** 1 Department of Medicine and Surgery, Internship Program in Geriatrics, Università Campus Bio-Medico di Roma, Via Alvaro del Portillo, 21-00128 Roma, Italy; s.zotti@unicampus.it (S.Z.);; 2 Operative Research Unit of Internal Medicine, Fondazione Policlinico Universitario Campus Bio-Medico, Via Alvaro del Portillo, 21-00128 Roma, Italy; 3 Operative Research Unit of Emergency Medicine, Fondazione Policlinico Universitario Campus Bio-Medico, Via Alvaro del Portillo, 21-00128 Roma, Italy; 4 Department of Medicine and Surgery, Research Unit of Geriatrics, Università Campus Bio-Medico di Roma, Via Alvaro del Portillo, 21-00128 Roma, Italy; 5 Operative Research Unit of Geriatrics, Fondazione Policlinico Universitario Campus Bio-Medico, Via Alvaro del Portillo, 21-00128 Roma, Italy

**Keywords:** bioelectrical impedance analysis, hand-grip strength, SARS-CoV-2, Mini-Nutritional Assessment, nutrition, in-hospital mortality

## Abstract

Hospitalized patients with respiratory failure due to SARS-CoV-2 pneumonia are at increased risk of malnutrition and related mortality. The predictive value of the Mini-Nutritional Assessment short form (MNA-sf^®^), hand-grip strength (HGS), and bioelectrical impedance analysis (BIA) was determined with respect to in-hospital mortality or endotracheal intubation. The study included 101 patients admitted to a sub-intensive care unit from November 2021 to April 2022. The discriminative capacity of MNA-sf, HGS, and body composition parameters (skeletal mass index and phase angle) was assessed computing the area under the receiver operating characteristic curves (AUC). Analyses were stratified by age groups (<70/70+ years). The MNA-sf alone or in combination with HGS or BIA was not able to reliably predict our outcome. In younger participants, HGS showed a sensitivity of 0.87 and a specificity of 0.54 (AUC: 0.77). In older participants, phase angle (AUC: 0.72) was the best predictor and MNA-sf in combination with HGS had an AUC of 0.66. In our sample, MNA- sf alone, or in combination with HGS and BIA was not useful to predict our outcome in patients with COVID-19 pneumonia. Phase angle and HGS may be useful tools to predict worse outcomes in older and younger patients, respectively.

## 1. Introduction

Malnutrition is highly prevalent in hospitalized older adults and may affect length of hospital stay and mortality [1,2]. Screening for malnutrition upon hospital admission is one of the crucial steps during an acute illness clinical evaluation and numerous studies have demonstrated that undernutrition may influence clinical outcomes in intensive care units [3]. This is particularly true in patients with lung diseases, presenting a complex and multifactorial interplay of systemic inflammation that leads to cachexia, decreased nutrition from poor intake, and reduced exercise tolerance from sarcopenia and dyspnea [4]. Indeed, in COVID-19 disease, the increased caloric needs due to proinflammatory cytokine storm induced by SARS-CoV-2, and the reduced food intake associated with dyspnea, cough, loss of taste and smell, and chronic asthenia, are additional risk factors for malnutrition [5,6,7,8]. In turn, in addition to other well-known aspects, pre-existing inadequate nutrition has been associated with SARS-CoV-2 disease severity [9].

This consideration may be even more evident in patients receiving high-flow oxygen and non-invasive ventilatory support, in whom oral nutrition is rather difficult and the infection’s course tends to be longer and more severe [10]. Moreover, the inflammatory process generated by the host in response to infection with acute respiratory failure leads to a disease-induced energy imbalance, promoting malnutrition and muscle wasting as well [11]. In fact, it is widely recognized in the literature that undernutrition negatively influences respiratory muscle function both through a direct effect on fiber size reduction, and indirectly, by inducing muscle composition derangements [11]. This may further lead to poor diaphragmatic and respiratory muscle function and to a prolongation of patients’ in-hospital recovery and discharge.

Several studies have previously compared different nutritional screening tools to find one that could better identify an increased risk for worse outcome and mortality in people with COVID-19. In a systematic review on people over 65 years of age with COVID-19, the Mini Nutritional Assessment short form (MNA-sf) was demonstrated to have the best predictive validity for poor appetite and weight loss over 2.6 kg as the Nutritional Risk Screening 2002 (NRS-2002) for the length of hospital stay (LOS) [12]. In another study with similar patients’ characteristics, MNA-sf was also associated to a longer LOS and heavier disease severity in those classified as malnourished according to the criterion of the nutritional risk screening itself, as well as NRS 2002 and the Nutrition Risk Index (NRI) [13]. Furthermore, in older people with COVID-19 infection, the presence of very low body mass index (BMI < 18.5) or MNA-sf scores indicating malnutrition (MNA-sf 0–7 points) was strongly related to a greater risk of in-hospital mortality, while surprisingly, no association was found with obesity [14].

In addition to this, the European Society of Clinical Nutrition and Metabolism (ESPEN) definition of malnutrition does not fully consider the loss of muscle function seen in patients with lung disease, that should be preferably monitored for pulmonary cachexia [4,15]. In clinical practice, a weight loss >5%, a weight <90% of ideal body weight, or a BMI ≤ 20 is commonly used to make a diagnosis of malnutrition. Otherwise, a more precise evaluation can be carried out using bioelectrical impedance analysis (BIA), that allows an inexpensive estimation of body composition and is feasible at bedside. Numerous works have demonstrated so far that BIA and muscular strength, measured using the handgrip strength (HGS) test, may be concomitantly used with basic nutritional parameters such as BMI and biochemical indicators to predict adverse events in critical illness [16,17]. Among all BIA-derived parameters, phase angle (PhA), reflecting the ratio of resistance to reactance expressed as an angle, is considered a biological marker of cellular health, as high cell mass and robust cell membranes cause delayed signals. A lower PhA has been correlated with increased mortality and length of hospital stay in various diseases [18,19]. In COVID-19, this parameter improves the discriminative power for worse outcome in addition to sex, age, and respiratory rate and is a significant predictor of mortality in patients admitted to intensive care units (UTI) [20,21,22]. Our hypothesis is that HGS and BIA, being economical and more precise, in addition to MNA-sf previously validated in a COVID-19 setting, could be used to better predict in-hospital complications related to a poor nutritional status in patients with acute respiratory failure due to SARS-CoV-2 pneumonia. Indeed, COVID-19 is expected to be a continuing healthcare problem despite mass vaccination [9] and identifying older patients having an inadequate nutritional status and an increased risk of adverse outcome may help clinicians to stratify those who need more intensive care.

Finally, Gómez-Uranga et al. [23] showed a significant relationship among nutritional and functional status following hospital discharge in older patients with COVID-19, apart from severe pneumonia. This finding highlights the importance of patients’ nutritional care and management during hospitalization, even for preventing loss of independence and the occurrence of new disability, which may influence some long-term consequences of SARS-CoV-2 infection.

The objective of the study was to assess the ability of HGS and BIA-derived body composition parameters in addition to MNA-sf in predicting in-hospital mortality or endotracheal intubation, in patients affected by a severe acute respiratory failure and admitted to a sub-intensive COVID-19 unit (SICU). 

## 2. Materials and Methods

### 2.1. Study Design and Participants

This observational retrospective study included 101 patients with SARS-CoV-2 infection who were consecutively admitted to the SICU of Campus Bio-Medico Hospital in Rome from November 2021 to April 2022. Viral infection was confirmed by a positive result of real-time reverse transcriptase polymerase chain reaction (RT-PCR) assay of nasal and pharyngeal swab specimens. The study protocol was followed in accordance with the tenets of the Helsinki Declaration and was approved by the Campus Bio-Medico University Ethical Committee in April 2020 (protocol code 22/20 OSS). A written informed consent form was signed for inclusion prior to the start of the study from all subjects. Clinical data, including age, sex, smoking habit, associated comorbidities (Charlson comorbidity index), and type of O2 therapy and pharmacological therapy started, were collected from the medical charts. In addition, CT severity score, a CT scan-based index, ranging from 0 (best) to 20 (worst), was used by our radiologists to visually quantify lobar regions affected by COVID-19 pneumonia. This scale was validated in a previous SARS-CoV-2-related study as well [24].

### 2.2. Nutritional Assessement and Outcome

The MNA-sf was performed as an initial screening test, as the score has a high negative predictive value for malnutrition [25]. This questionnaire, including 6 items rated between 0 and 3 relative to aspects of the subject’s nutritional intake, can range from 0 to 7, 8 to 11, or 12 to 14, suggesting that the person is malnourished, at risk, or has a normal nutritional status. A validation study demonstrated good sensitivity compared with the full MNA [26]. At admission, weight was measured to the nearest 0.1 kg using a high-precision mechanical scale and standing height to the nearest 0.1 cm based on wall measure with participants wearing light indoor clothes and no shoes. When this was not possible, due to the clinical conditions of the patients, the reported height and weight were recorded. These values were then used to calculate the body mass index (BMI, kg/m^2^) and routine biochemistry exams were collected as well.

### 2.3. Body Composition and Muscular Strenght

The European Working Group on Sarcopenia in Older People (EWGSOP) stated that sarcopenia is defined as low skeletal muscle mass and reduced muscle function [27]. Furthermore, muscle wasting in severe pneumonia, as in COVID-19, is likely due to a mixture of poor nutrition, deconditioning, systemic inflammation, and sometimes medication (e.g., oral glucocorticoids). Accordingly, HGS (Hydraulic Dynamometer, RO  +  TEN, Milan, Italy) and bioelectrical impedance analysis (BIA101, Akern, Firenze, Italia) were obtained in this study to estimate participants’ muscular strength and mass, respectively. Considering BIA-derived parameters, skeletal mass index (SMI, calculated by the equation as skeletal muscle mass divided by height squared, SMM/height2) [28] and phase angle (PhA, calculated by measuring the resistance (R) and reactance (Xc) after the bioimpedance applied current) [29] were selected for this study. Abnormal HGS and SMI were evaluated when lower than 27 kg and <16 kg, and <7 kg/m^2^ and <5.5 kg/m^2^, for males and females, respectively, according to the EWGSOP criteria [27]. These cut-off points result from the available sarcopenia literature, based on normative populations or predictive populations when data were unavailable. Although PhA was not present in the EWGSOP measurable variables, it was shown in previous studies to be a good indicator of malnutrition and sarcopenia [30,31]. It was therefore included in these analyses and was considered pathological when <6° or >10°, according to a previous study found in the literature [32].

### 2.4. Outcome

Outcome was defined as death or as a transfer to the intensive care unit (ICU) for endotracheal intubation during hospitalization in SICU. 

### 2.5. Statistical Analysis

Since the baseline risk of and risk factors for adverse outcome differ between younger and older patients [33], analyses were stratified by age group (<70, ≥70 years). Continuous variables were expressed as mean and SD, and categorical variables as absolute numbers and percentages. The performance of MNA-sf and HGS with the addition of BIA parameters in predicting the occurrence of the outcome was assessed using receiver operating characteristic (ROC) curves. Statistical analysis was performed using R software version 4.2.2. 

## 3. Results

### 3.1. Patients’ Characteristics 

Overall, 101 patients with SARS-CoV-2 pneumonia who were hospitalized in SICU were included. The mean age was 69.2 (SD 13.2) years; 61 (*n* = 60%) were males (Table 1).

As shown in Table 1, the mean Charlson comorbidity index score was 4.3 (SD 2.9) and it was higher in the older group, as expected. In the study population, 39% had a smoking habit with a relatively superior percentage in the group of patients ≥70 years old (40%).

### 3.2. Respiratory Support

In this sample, 43% (*n* = 44) needed non-invasive ventilation (NIV) or continuous positive airway pressure (CPAP); the remaining 54% (*n* = 55) received oxygen supplementation through high-flow nasal cannula (HFNC). No significant differences in the oxygen support used during acute respiratory failure was found among the two groups.

Respiratory failure was moderate–severe in both study samples with an average PaO2/FiO2 ratio of 122.7 (SD: 53.9). Similarly, the severity score of parenchymal infection involvement evaluated by chest computed tomography scan (CT) was on average 12.4 out of 20 (SD: 4.4), with no relevant variation according to patients’ age group. Overall, 36 patients (*n* = 35%) were transferred in the ICU for endotracheal intubation or died during our hospitalization. As expected, a twofold percentage of patients older than 70 years had an adverse event compared to younger ones.

### 3.3. Nutritional Parameters and Muscle Assessment

As shown in Table 1, participants were on average overweight with a mean BMI of 29.7 Kg/m^2^ (SD: 7.3), while the total protein serum concentration was 6 g/dL (SD: 0.7). The MNA-sf average score was 10.3 (SD: 2.2) with 40% and 60% of patients being categorized as having normal nutritional status and being at risk for malnutrition, respectively. The risk of malnutrition was higher in older participants (75%, *n* = 43) compared to younger adults (31%, *n* = 14) and reduced HGS was found in 13 older patients and in 2 younger ones. Body composition showed normal SMI in all participants, while 51 older participants and 36 younger ones had a reduced PhA, respectively. 

### 3.4. Prediction of Clinical Outcomes

Overall, MNA-sf had a fair sensitivity (0.70) and a poor specificity (0.45) for outcome discrimination, with an AUC of 0.6. The MNA-sf predictive ability did not significantly change with the addition of HGS (AUC: 0.66) and BIA-derived parameters such as SMI (AUC: 0.59) and PhA (AUC: 0.60).

As shown in Table 2, after stratification by age in younger participants, the MNA-sf had an AUC of 0.59, and the addition of BIA-derived parameters to the MNS-sf did not show any significant results (Table 2).

**Figure 1:**
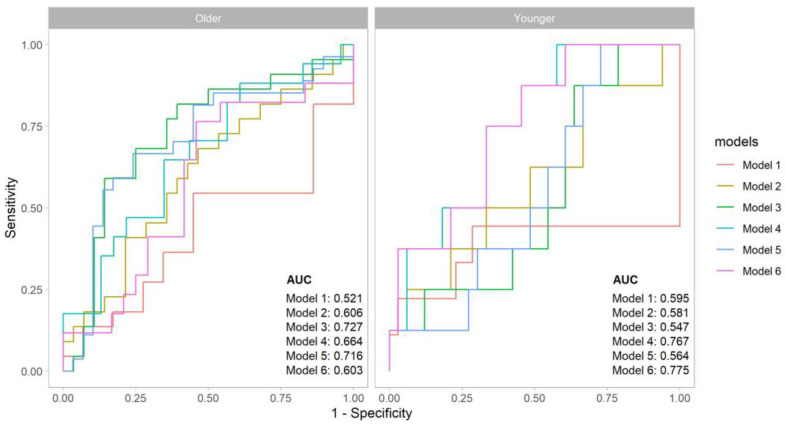
Comparative analysis of ROC curves for the outcome in the two age groups. Legend: Model 1 for MNA-sf; Model 2 for MNA-sf + SMI; Model 3 for MNA + PhA; Model 4 for MNA + HGS; Model 5 for PhA; Model 6 for HGS.

In addition, the combination of MNA-sf and HGS did not have a better predictive ability compared to the HGS alone (AUC: 0.77, Figure 1).

On the contrary, the sole HGS revealed a good predictive ability of adverse event with an AUC of 0.77, a sensitivity of 0.87, and a specificity of 0.54 (Table 2).

In the older population, the MNA-sf had a poor outcome discrimination (AUC 0.52), while PhA demonstrated a greater sensitivity and specificity with an AUC of 0.72. Similar results were found with the combination of MNA-sf and PhA (AUC: 0.73). The SMI also did not reveal a significant discriminative ability (Table 2). Finally, in contrast with the findings in the younger people, the AUC increased from 0.60 to 0.66 when MNA-sf was considered together with HGS values (Figure 1).

## 4. Discussion

In our population, the MNA-sf, with or without the addition of grip strength or indicators of body composition, was not able to adequately discriminate the risk of mortality or ETI during COVID-19 pneumonia. The parameters that could better predict the outcome were the PhA and the HGS in older and younger participants, respectively.

Our results are in line with a previous Italian study conducted in an internal medicine ward, that showed that PhA was effective in predicting the occurrence of worse clinical outcomes compared to other BIA-derived parameters that did not add a further predictive value (AUC of 0.50, 0.52 and 0.57 for fat mass, fat-free mass, body cell mass, respectively) [34]. In this study, the PhA revealed an AUC of 0.597 (CI 95%, 0.486–0.708) for the composite outcome of death and admission in an ICU and of 0.589 (CI 95%, 0.483–0.696) for prolonged hospitalization, with a sensitivity of 82% and a specificity of 45%. Our results provide more convincing evidence that PhA is a good BIA-derived parameter that can predict disease severity in older patients [18,19]. Geng et al. [35] showed that PhA, an indicator of cellular health that is negatively associated with age, is a useful bioelectrical marker for skeletal muscle quantity and quality in hospitalized elderly patients. Indeed, they underlined how the decline in skeletal muscle mass may occur concurrently with an increase in fat infiltration and lead during aging to a decrease in reactance, an increase in resistance, and associated lower PhA values. Changes in the number and function of skeletal muscle fibers and consequent impaired muscle contractility and decreased muscle strength could even impact on respiratory muscles, and affect the work of breathing and respiratory mechanics in older patients [36]. This hypothesis may partially explain why PhA lost its discriminative power of adverse events during severe acute respiratory failure in the younger group. Since older patients have revealed to be at higher risk of the COVID-19 disease severity, even after the advent of vaccination, PhA analyses may easily help to stratify the risk of adverse outcomes at admission in this peculiar population.

In addition, after stratification for age, our results indicate that HGS has a satisfying predictive power in patients younger than 70 years and a modest discriminative ability in older patients when combined with MNA-sf. These data agree with a previous study on COVID-19 showing that a pre-existing condition of reduced HGS may increase the vulnerability to COVID-19 and aggravate disease severity leading to ICU admission, the need for mechanical ventilation, and mortality [37]. Indeed, this finding suggests that in hospitalized younger patients for COVID-19 pneumonia and respiratory failure, measurement of HGS, representing an observable loss of muscle strength, could serve as an inexpensive and feasible tool to early identify patients at higher risk of complications and adverse prognosis. As shown in Table 1, the HGS values’ range was wider in younger patients, and this reason may partially interpret the lower discriminative ability of HGS for adverse events in the older group.

Although we found a denoting divergence among the “age-related” groups, the assessment of both muscular function and body composition represents a strength of this study. Indeed, the results of this prognostic analysis underline the need for using both muscle mass and muscle strength evaluation in the diagnosis of sarcopenia as EWGSOP recommendations suggest. This is explained by the fact that the relationship between muscle mass and function is not linear and the assessment of only one variable may be of limited clinical value. As an example, it could have underestimated the diagnosis of “pre-sarcopenia”, characterized by low muscle mass without impact on muscle strength or physical performance. Similarly, in our study sample, it would have omitted those younger patients presenting a poor physical performance status as measured by HGS with non-pathological PhA or SMI values. Recognizing different stages of sarcopenia and predicting a poor prognosis at distinct ages may help in selecting treatments and setting appropriate recovery goals, even during an acute illness, as in COVID-19 disease.

Our study has some limitations. First, due to the challenging conditions of the COVID-19 pandemic, the number of patients included in the analysis was rather small and it was a single-center study. Second, performing the MNA-sf questionnaire and HGS required patient collaboration and did not permit to include in the sample study all those patients affected by severe cognitive disorders or those who were sedated to permit NIMV compliance (e.g., dexmedetomidine) that were not able to collaborate. Another limitation of the study was that we did not conduct a dynamic control of patients’ caloric intake during the hospitalization, and we did not verify any effect of nutritional interventions. Furthermore, it was not possible to differentiate whether poor nutrition was due to a chronic condition or was induced by the acute disease. Finally, statistical analysis was not adjusted for any intra-hospital complications’ development, such as infections or cardiovascular events and/or causes of death, since this information was not present in our dataset.

Despite all these limitations, integrating BIA and HGS as a nutritional assessment at admission revealed to be economical and easily accessible, and could permit to focus on undernourished patients with COVID-19, other than disease severity. In the context of systemic inflammation induced by SARS-CoV-2, patients with malnutrition could enter a vicious cycle that may predispose them to an increased risk of complications and adverse events. Indeed, COVID-19 disease, in terms of acute infections in general, not only includes respiratory symptoms but also all those features leading to an insufficient nutritional intake such as loss of appetite, gastrointestinal disturbances, and chronic fatigue. This aspect could further worsen patients’ nutritional status [5,6,7,8]. Therefore, this approach may help clinicians to further evaluate those having an in-hospital reduced caloric intake, who have been shown in the literature to have a significantly increased risk of mortality [38].

Furthermore, while the exact pathogenesis of malnutrition in pulmonary diseases remains unclear, it seems likely that factors contributing to energy imbalance include changes in metabolism and caloric intake, disuse atrophy, tissue hypoxia, inflammation, and medications [39]. This syndrome, called pulmonary cachexia, is associated with an accelerated decline in functional status and mechanical abnormalities (e.g., airflow limitation and lung stiffness) leading to increased work required for breathing and an occurrence of respiratory failure with lesser degrees of respiratory muscle weakness [4,15]. Collectively, this information supports the clinical role of assessing complementary muscle function and mass during respiratory disorders at hospital admission, even for prognostic purposes. Thus, malnutrition may be further associated with progressive diaphragmatic weakness leading to the worsening of respiratory failure, an effect that in patients with pneumonia, as in COVID-19, could even be exacerbated.

In conclusion, patients hospitalized for COVID-19 pneumonia, malnutrition, sarcopenia, and frailty showed to be interrelated entities, especially in patients with greater baseline functional impairment prior to admission [23]. Thus, rather than disease severity, the effect of reduced nutritional intake during hospitalization for acute SARS-CoV-2 infection, may have a negative impact on post-discharge functional autonomy or trigger the occurrence of a new disability. The continuing high prevalence of SARS-CoV-2, even after mass vaccination along with the growing interest in post-acute COVID-19 syndrome increase the need to apply these results in new studies and research. Therefore, identifying on-time patients requiring additional nutritional care during hospitalization may permit the management of all nutritional modifiable risk factors, such as correcting daily diets, and prevent older people’s functional status from worsening, institutionalization, and death.

## 5. Conclusions

This study lends support to the use of BIA in very old patients, and HGS primarily in younger patients, who are receiving non-invasive or high-flow oxygen support during COVID-19 infection or for any clinical illness, as prognostic indices to possibly evaluate disease severity and adverse events. New studies need to be conducted to monitor patients’ clinical evolution and caloric intake during hospitalization in other non-COVID-19 units and verify any effect of nutritional interventions that may also influence the outcome. A proper identification of malnourished patients, who are admitted for respiratory failure and who require respiratory support as NIMV or HFNC, may allow clinicians to include nutritional support in addition to standard medical therapy to begin during hospitalization. In these patients, active nutritional supplementation could improve respiratory muscle function and exercise performance during respiratory rehabilitation programs, and possibly ameliorate the respiratory mechanics, compliance to the diverse respiratory supports, and promote their weaning.

## Figures and Tables

**Table 1:** Baseline characteristics of all patients (*n* = 101).

Variables as Mean (SD)	All Patients *n* = 101	<70 Years Old *n* = 44	≥70 Years Old *n* = 57
Age (years)	69.2 (13.2)	56.7 (9.0)	78.8 (5.8)
Sex (male) (%)	60	65	56
BMI (Kg/m^2^)	29.7 (7.3)	31.7 (8.5)	28.2 (5.9)
Smoking habit (%)	39	25	40
CCI	4.3 (2.9)	2.1 (2.0)	6.1 (2.2)
PaO_2_/FiO_2_	122.7 (53.9)	121.3 (48.5)	123.7 (58.1)
Chest CT SS	12.4 (4.4)	12.7 (4.5)	12.1 (4.3)
NIV or CPAP (%)	43	52	60
HFNC (%)	54	47	40
MNA- sf^®^	10.3 (2.2)	11.1 (1.7)	9.6 (2.3)
Total protein (g/dL)	6 (0.7)	6.3 (0.7)	5.8 (0.6)
HGS	27.5. (12.7)	33.8 (12.9)	21.2 (9.0)
SMI (SMM//height^2^)	10.6 (2.2)	11.1 (1.9)	10.2 (2.3)
PhA	4.5 (1.7)	5 (1.8)	4.2 (1.5)
Death—ETI	35%	20%	47%

Abbreviations: BMI, body mass index, CCI, Charlson comorbidity index; chest CT SS, chest computerized tomography severity score; NIV, non-invasive ventilation; CPAP, continuous positive airway pressure; HFNC, high-flow nasal cannula; MNA-sf^®^, Mini Nutritional Assessment short form; HGS, handgrip strength test; SMI, skeletal muscle index; PhA, phase angle; ETI, endotracheal intubation.

**Table 2:** Variable models for the outcome in younger and older participants.

**Models in Participants < 70 Years**	**Sensitivity**	**Specificity**	**AUC**
MNA-sf^®^	0.22	0.97	0.59
PhA	1	0.27	0.56
SMI HGS	0.5 0.87	0.79 0.54	0.59 0.77
MNA-sf^®^ + PhA	0.87	0.36	0.54
MNA-sf^®^ + SMI	0.87	0.33	0.58
MNA-sf^®^ + HGS	1	0.42	0.77
**Models in Participants ≥ 70 years**	**Sensitivity**	**Specificity**	**AUC**
MNA-sf^®^	0.54	0.55	0.52
PhA	0.66	0.76	0.72
SMI HGS	0.63 0.76	0.62 0.54	0.58 0.60
MNA-sf^®^ + PhA	0.59	0.86	0.73
MNA-sf^®^ + SMI	0.68	0.53	0.60
MNA-sf^®^ + HGS	0.65	0.65	0.66

Abbreviations: MNA-sf^®^, Mini Nutritional Assessment short form; HGS, handgrip strength test; SMI, skeletal muscle index; PhA, phase angle.

## Data Availability

The data presented in this study are available on request from the corresponding author.
